# Gene Duplication and Ectopic Gene Conversion in *Drosophila*

**DOI:** 10.3390/genes2010131

**Published:** 2011-02-11

**Authors:** J. Roman Arguello, Tim Connallon

**Affiliations:** Department of Molecular Biology and Genetics, Cornell University, 107 Biotechnology Building, Ithaca, NY 14853, USA; E-Mail: tmc233@cornell.edu

**Keywords:** ectopic gene conversion, gene conversion, non-allelic conversion, gene duplication, *Drosophila*, selection

## Abstract

The evolutionary impact of gene duplication events has been a theme of *Drosophila* genetics dating back to the Morgan School. While considerable attention has been placed on the genetic novelties that duplicates are capable of introducing, and the role that positive selection plays in their early stages of duplicate evolution, much less attention has been given to the potential consequences of ectopic (non-allelic) gene conversion on these evolutionary processes. In this paper we consider the historical origins of ectopic gene conversion models and present a synthesis of the current *Drosophila* data in light of several primary questions in the field.

## 1. Introduction

Gene duplication is a central theme in evolutionary genetics due in large part to each duplicate's potential for introducing genetic novelty. Gene family expansions have jointly contributed to genome size [1], and to the diversification of molecular functions, including those influencing morphology [2], digestion [3–5], immune defense [6], and possibly reproductive isolation between species [7–9]. While there is evidence for adaptive differentiation between duplicates [10–12], duplication events can also have deleterious consequences, by generating chromosomal instability and dosage abnormalities [13–16]. As a result, research on gene duplication is of both evolutionary and medical interest.

**Figure 1:**
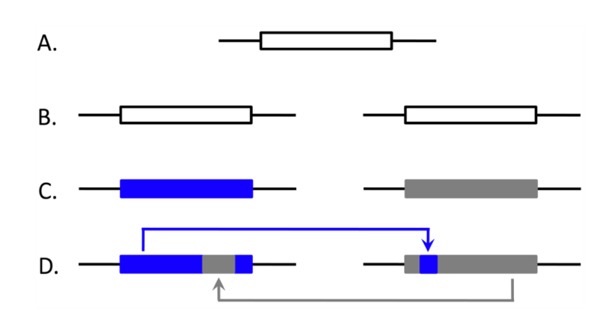
A graphical model of gene duplication and paralog evolution with EGC. An ancestral single-copy gene (**A**) becomes duplicated, leading to initially identical paralogs (**B**). Independent accumulation of substitutions will lead to paralog divergence from the ancestral sequence and differentiation between paralogs ((**C**) with white, blue and gray representing divergent sequences). EGC events re-homogenize the sequences ((**D**) with two conversion tracts shown), with substitutions from one duplicate being shared by the other.

Though most duplicate alleles will eventually be lost from a population, a complex interaction between genetic drift, mutation and selection can occasionally lead to duplicate fixation and preservation [17]. Unlike single-copy genes, paralogous (or “non-allelic”) genomic regions can interact via ectopic (non-allelic) gene conversion (EGC). Gene conversion refers to a double-strand break (DSB) induced form of homologous recombination, with EGC occurring between paralogous regions with high sequence identity. The mechanism results in the transfer of a chromosomal region from the intact sequence to the region that contains the DSB, and can occur between homologous or nonhomologous chromosomes. From an evolutionary or population genetic perspective, this is often modeled as a “copy and paste” process of nonreciprocal exchange ([18]; Figure 1), which introduces genetic interdependence between duplicates and partially governs their evolutionary fates [19]. Despite its name, EGC does not occur exclusively within genes (it can occur in noncoding sequences).

A large body of theoretical work illustrates that EGC can greatly influence the evolutionary dynamics of duplicates [17,19], yet empirical support of the theory, including its effect on the process of adaptation and gene family evolution, is less clear. In this article, we present a review and synthesis of the empirical literature on EGC as it pertains to *Drosophila*. After summarizing commonly used methods for detecting EGC, the paper is structured into two main sections. In the first, we briefly outline the historical context in which EGC came to be studied in *Drosophila*, and describe how EGC research emerged from a more general analysis of repetitive DNA and concerted evolution. The second section provides an up-to-date analysis of the Drosophila empirical literature concerning duplication and EGC. We anchor the synthesis around several broad and unresolved questions:

- What is the relative contribution of EGC to patterns of concerted evolution?
- How does genomic context (e.g., nucleotide base composition; linkage relationships between duplicates) affect EGC?
- How do selection and EGC interact to influence adaptation?
- Does gene conversion between duplicates bias estimates of the gene duplication rate and the tempo of paralog differentiation?

Our general conclusion is that EGC, at minimum, plays a consequential role during the early evolution of physically linked *Drosophila* duplications. However, the empirical limitations of jointly testing for interactions between conversion, selection, linkage, and gene family size preclude a strong conclusion about the temporal duration of conversion between duplicates, or its role in promoting or constraining adaptation. We describe future analyses that may shed light on these unresolved issues.

## 2. Detecting Ectopic Gene Conversion

**Figure 2:**
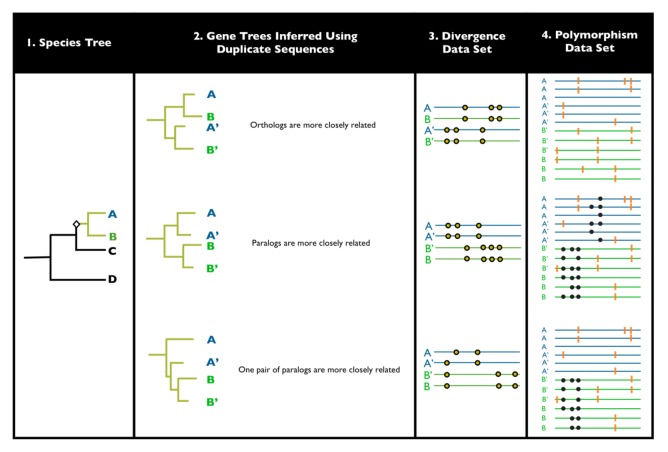
Schematic illustration of data that can be used to detect EGC. *Panel 1*: A species tree for 4 arbitrary species with a single gene duplication event noted by the black diamond. Green tree tips indicate that the branches leading to species A and B carry the gene duplication. Species A and B are considered in the following panels, while C and D are ignored. *Panel 2*: Three examples of gene trees inferred from the paralog sequences. The duplicate copy is noted by the apostrophe. The top gene tree is expected if there is no EGC, while the bottom two trees may arise if there is gene conversion between one (bottom tree) or both paralog pairs (middle tree). *Panel 3*: Hypothetical divergence data sets associated with the gene trees from panel 2. Circles refer to molecular markers (e.g., nucleotide substitutions or restriction sites) that are fixed between orthologs or paralogs. Markers that align vertically are shared between sequences. *Panel 4*: Hypothetical polymorphism data sets associated with the gene trees from panel 2. Black circles indicate shared polymorphisms between paralogs and orange lines indicate unshared (“private”) polymorphisms between paralogs. See the text for additional information.

Methods for detecting EGC have been developed for both divergence- (multi-species alignments of singly-sampled paralogs) and polymorphism-based sequence data sets (multiple alignments, per paralog, per population). Though the scope of this review does not include a detailed discussion of the methods used, it is helpful to introduce and highlight the most commonly used approaches for detecting EGC (Figure 2). Throughout the paper, we also highlight some of their limitations, when these are directly applicable to interpreting the data.

Within the *Drosophila* literature, the two most cited divergence-based approaches utilize the GENCONV software package [20] or test for incongruities between a given species phylogeny and a gene tree that has been estimated from paralogous and orthologous DNA sequences from one or more of the same species represented in the phylogeny. GENCONV was originally designed to detect allelic conversion, but has subsequently been used to detect EGC. The software searches for stretches of sequence identity between duplicates (tracts) that extend further than would be expected by chance, given a model of independent evolution between the loci. Permutation tests are used to establish statistical significance [20].

Tests of incongruity between species trees and gene trees are based on the following logic. If phylogenetic information suggests that a given duplication event preceded speciation between two or more species, but DNA sequence data for paralogs within species demonstrate greater sequence identity than orthologs between species, then the datasets are identified as “irreconcilable”. In these cases, EGC can be invoked to explain the disagreement between phylogenetic dating of duplication events and the relative sequence identity between paralogs and orthologs (e.g., [21]). Such reasoning can be extended to gene trees constructed from different sub-regions of a duplicate sequence, where variation between sub-regions can be used to identify conversion tracts (e.g., [22]). Such tests between gene trees and species trees will subsequently be referred to as “reconciliation” methods.

A third divergence-based method is based on analysis of two types of nucleotide substitutions between paralogous and orthologous sequence alignments: 1) substitutions between orthologs that are shared between paralogs; and 2) substitutions between paralogs that are shared between orthologs. The former pattern supports a conversion model, while the latter is indicative of evolutionary independence between paralogs [22]. Through parsimony-based arguments, one can test a hypothesis of EGC by calculating the probability of observing the data for each substitution type, given a null model that permits multiple mutations but no conversion. We refer to this as the “site-specific” method.

The least widely used (but most powerful) method relies on polymorphism data within a species. Alignments of the set of paralogs can be used to identify shared polymorphism. Given a low point mutation rate (as expected), parallel mutations and shared polymorphism will be rare without EGC. The actual amount of shared polymorphism between paralogs can be used to identify recent conversion events, and to estimate the rate of conversion between the paralogs [23]. We refer to this approach as the “shared polymorphism” method.

## 3. Historical Background to Ectopic Gene Conversion

### 3.1. Repetitive DNA and the Origin of Genome Size Variation

Current debates about EGC can be traced to earlier ones over concerted evolution from the mid 1960s to the 1970s. These debates, in turn, were intertwined with emerging interest in the evolution of genome size and the underlying importance of repetitive DNA.

The first empirical evidence for genome size plasticity traces back to the *Bar* locus duplication, discovered by members of the Morgan lab [24–25]. During the interval between the discovery of the *Bar* duplication and the first evidence of concerted evolution in 1972 [26], advances in microscopy, chromosomal labeling, and DNA and RNA hybridization and denaturation methods, revealed striking variability in genome size [27–28]. Along with these observations came speculation over the mutational and evolutionary mechanisms driving genome size differences. Modern models of unequal crossing over and replication error were hypothesized as the primary mechanisms generating genome content differences [24,29–32], while subsequently discredited models, such as variability in the number of strands comprising chromosomes, were also considered at the time [31,33]. In addition to uncertainty surrounding duplication mechanisms, cytogeneticists found that the existence of multiple gene copies presented a significant challenge to the way chromosomes were conceptualized [32].

From this earlier research on genomic content, the model most relevant to ectopic conversion is the master-slave model [31,34]. Working with the giant lampbrush chromosomes of female newts, Callan and Lloyd [34] measured chromosome loop sizes within and between two subspecies. They argued that recombination took place within them, and that the loop morphologies were genetically determined and followed Mendelian segregation. They reasoned that the content of each loop was comprised of a series of identical, repetitive DNA units [31]. Such an observation was relevant to genome size differences, as variability in repeat copy number could contribute to the variation [34]. That a large number of functionally important repetitive units represented a large mutational target did not go unnoticed, and the master-slave model was introduced to explain how sequence identity between repeats could be maintained in the face of mutation [31,34]. As a predecessor to current models of (biased) EGC, this early model invoked a single “master” and a series of “slave” copies within each repeat family. Following meiotic recombination, each slave copy would pair with the master and become homogenized exclusively in the master-to-slave direction. Thus, all mutations accumulating in slave copies would be “rectified” to their ancestral state (master copies were assumed to be largely protected from mutation accumulation).

Later modifications to the “rectifying” model attest to its appeal during this time. For example, the original master–slave model assumed that each master gene was separated by intervals comprised of repetitive slave copies, yet subsequent data indicated that unique genes (rather than copies) were likely to be physically linked [32]. Thus, the “Cycloid Model” (in response to the emerging linkage data) proposed that a loop of slave genes became detached from the main chromatid prior to a cross over, and these genes were subsequently reinserted following homogenization with the master (for additional master–slave based physical models see [35]). Around the same time, Edelman and Gally [36–37] proposed a “democratic” gene conversion model, which invoked symmetrical exchanges (non-biased, as in the master–slave) between repeated genes. The democratic model forwarded arguments that selection could act more efficiently when a beneficial mutation arising on any gene copy was free to spread throughout the gene family.

By the start of the 1970s, much emphasis had been placed on understanding the origins and implications of repetitive DNA, which raised questions about the evolutionary maintenance of repeats, conversion biases, interactions between selection and conversion, and the importance of chromosomal context in mediating concerted evolution. These questions remain important today.

### 3.2. Concerted Evolution, rDNA, and Drosophila

To improve understanding of the evolutionary dynamics of repetitive DNA, the pre-molecular biology world needed a more tractable model system. rDNA was well suited for this role [38,39], and served as an early model for duplication and conversion in *Drosophila*.

The abundant transcriptional products of rDNA loci, together with hybridization and denaturation methods of the time, provided an opportunity to estimate copy number differences and sequence divergence of repetitive units within and between species. Detailed evolutionary studies of rDNA arrays across diverse taxa gained momentum starting in the mid 1960s [28]. Most notable is that of Brown *et al.* [26], which provided the first example of concerted evolution, using closely related *Xenopus* species. They showed that individual rDNA array units exhibited very high sequence identity within compared to between species. This suggested that a “correction mechanism” between repeats resulted in “horizontal evolution” within species. Though Brown *et al.* [26] did not initially refer to this observation as “concerted evolution”, the pattern has subsequently been referred to as such.

With evolutionary models for repetitive DNA already developed, the field was situated to integrate empirical patterns of concerted evolution. One explanation for the pattern invoked the already popular model of unequal crossing over. High rates of unequal crossing over could permit the stochastic spread of identical copies throughout a given array, leading to a pattern of high sequence identity between individual copies. The alternative explanation harkened back to models of homogenization between duplicates, such as the master–slave model. Though not conclusive, initial support for unequal crossing over was provided by *Xenopus* DNA data [26,40], and was reinforced by mathematical theory [41,42].

It had already been established that *Drosophila* had large rDNA arrays on both the X and Y chromosome. A role for unequal crossing over during the evolution of these arrays had been suggested from studies of the *bobbed* mutant, which was associated with deficiencies of X-linked rDNA genes [43]. Subsequent genetic analysis of the *bobbed* phenotype led to the discovery of “DNA magnification”, where male germlines deficient for both X- and Y-linked rDNA could revert to the wild-type rDNA gene number [39,44–46]. Though Ritossa [44] argued against the role of unequal crossing over in DNA magnification, subsequent work provided evidence that it occurred through unequal crossing over between sister chromatids during meiosis [47]. In addition to strain-specific (or germline) gains and losses of rDNA genes, signals of concerted evolution were also uncovered in rDNA arrays. This was first documented through comparative studies within the *D. melanogaster* species subgroup and *D. hydei* [48,49]. These studies provided two important insights. First, repeat units on nonhomologous chromosomes were shown to be capable of concerted evolution. Second, and in contrast to *Xenopus*, high sequence identity between repeats was found for the nontranscribed regions (it was later shown that homogenization of nontranscribed regions did not persist between distantly related *D. hydei* and *D. melanogaster*; [49]).

Following these early rDNA studies, patterns of concerted evolution have consistently been reported for this gene family [50]. While unequal crossing over and purifying selection were both thought to contribute to the homogenization of array units [51–53], there is currently less convincing evidence regarding a role for EGC [53].

## 4. Distinguishing Ectopic Gene Conversion and Gene Turnover as Drivers of Concerted Evolution in *Drosophila*

Debates over the role of EGC in generating patterns of concerted evolution in *Drosophila* have persisted to the present. The current form of the debate is strikingly similar to what one finds in the early concerted evolution literature, where competing hypotheses included homogenizing and “expansion-contraction” processes. Today, the debate is often framed as a contrast between EGC and “birth-and-death” models [54,55]. The birth-and-death model invokes the continuous generation of duplicate genes, with the rate of origin balanced by a steady rate of gene loss by pseudogenization or deletion. Assuming that duplicates rarely evolve novel functions (for which individual gene copies might be maintained by selection), then gene copy turnover will cause young duplicates to gradually replace older copies, in a process analogous to the neutral theory of molecular evolution (e.g., steady, clock-like replacement of older alleles with younger, neutral substitutions). Birth-and-death is expected to be most common in multigene families with members exhibiting variable degrees of divergence, relatively high sequence identity within gene families, and pseudogenes [55]. Though the name of the model is relatively recent, it is conceptually similar to concepts developed during the 1960s and 1970s (see above).

### 4.1. Ectopic Conversion from a Case Studies Perspective

There is little doubt that gene turnover and EGC both occur in *Drosophila*, and their relative contributions to patterns of concerted evolution are expected to vary on a case-by-case basis, as we describe below. Because of their idiosyncrasies, case studies have limited ability to address genome-wide frequencies of EGC and gene turnover. As such, we consider specific properties of gene families that make them susceptible to processes of EGC and turnover, and outline some experiments that will be necessary to better resolve the issue.

#### Heat Shock Proteins (HSP)

As an ancient and evolutionarily conserved gene family, HSPs are good *a priori* candidates for sustained EGC over relatively long timescales (paralogs are expected to have high sequence similarity due to concordant purifying selection). In the *D. melanogaster* species group, there are two pairs of HSP genes (*Hsp70Aa/Hsp70Ab* and *Hsp70Ba/Hsp70Bb*), with each pair tightly linked in a “palindromic” (*i.e.*, mirror image) orientation on the Muller E chromosome (chromosome 3R *of D. melanogaster*). This type of orientation has an interesting property with respect to ectopic recombination. A double-strand break in one of the paralogs can become resolved by gene conversion (resulting in sequence homogenization; e.g., Figure 1), or by crossing over, which can generate deleterious chromosomal abnormalities (inversions, and large-scale duplications and deletions [16]). Deleterious haplotypes caused by ectopic crossovers should contribute marginally to evolution, whereas conversion resolution of double-strand breaks will contribute to concerted evolution [16]. Thus, a palindromic orientation may minimize the generation of copy number polymorphism and evolution under a birth/death process. Two lines of evidence support ongoing conversion within each paralog cluster of HSPs. First, despite the relatively ancient origin of duplicate pairs (each precedes species divergence), paralogs within each species have higher sequence identity than ortholog pairs between species, consistent with ongoing EGC within the Drosophila lineages [21]. Second, paralogs share silent polymorphisms, indicating recent conversion events [56]. Recent work [57] also reports concerted evolution between a young set of rapidly evolving Hsp70 cofactor paralogs: *Hsc/Hsp70-interacting protein* (*HIP*). *HIP*/*HIP-R* genes are X-linked, non-inverted duplicates that are confined to the *D. melanogaster* lineage. Like HSP genes, extensive shared polymorphisms indicate that these cofactor duplicates are undergoing conversion.

#### Amylase

Another classic study system for *Drosophila* EGC is the amylase gene clusters. The *D. melanogaster* species group includes a conserved set of linked paralogs (*Amy-p* and *Amy-d*) in palindromic orientation (similar to *Hsp70* genes). Early work based on restriction site analysis [58] showed the widespread sharing of substitutions between paralogs. Inference of EGC was reinforced by subsequent, sequence-based analysis, which showed high intraspecific sequence identity between paralogs relative to divergence between orthologs [59,60], with the pattern of concerted evolution confined to coding sequence (flanking regions appear to evolve independently; [61,62]).

Subsequent work was extended to species outside of the melanogaster group, which carried an amylase cluster orthologous to *D. melanogaster*, and one or more additional clusters [63]. Concerted evolution between amylase paralogs within *D. pseudoobscura* inversion karyotypes were once again limited to coding regions [64,65]. *Drosophila kikkawai* and close relatives have two highly-divergent clusters of linked, palindromic amylase genes (*Amy1*/*Amy2* and *Amy3*/*Amy4*), with each cluster showing high sequence identity [66]. Homogeneous coding and noncoding sequence between *Amy3*/*Amy4* may indicate EGC or a duplication event. The *Amy1*/*Amy2* pair, which appears to be orthologous to the *melanogaster* cluster, shows evidence of coding (but no noncoding) concerted evolution, which supports a model of continuous EGC [67,68].

In many ways, these classical studies are representative of the case study approach to concerted evolution. Evidence for EGC is typically associated with the analysis of small gene families that are physically linked and/or evolving under purifying selection (e.g., larval cuticle protein cluster genes [69]; *Idgf* genes [70]; esterases [71,72]; histones [73]; HSP and amylase genes (see above)). High sequence similarity due to recent origin or sustained purifying selection, and tight linkage between paralogs, is expected to maximize opportunities for EGC. While neutral divergence at synonymous sites is certainly possible, purifying selection across a majority of coding sequence (nonsynonymous sites), coupled with relatively moderate levels of EGC can easily overwhelm divergence by genetic drift. It may therefore be unsurprising that these case studies provide the best evidence for ongoing EGC.

This is not to suggest that these criteria are necessary for EGC. Immunity and reproductive-related genes are expected to have elevated opportunities for diversification (their sequences often exhibit modest to low constraint; [6]), yet these types of genes exhibit clear patterns of EGC [74,75]. While analysis of linked *versus* dispersed loci supports the prediction that EGC rates are negatively correlated with the physical distance between paralogs [76–78], polymorphism-based data suggests that inter-chromosome interactions can persist between paralogs despite their distance and in the face of differential positive selection between paralogs [79].

On the other hand, a focus on small gene families will likely minimize the effect of gene turnover. Larger gene families are expected to be more permissive to the fixation of duplicates relative to smaller gene families because their sensitivity to deleterious dosage effects might be relatively low and their rate of copy number mutations might be relatively high. Consequently, the importance of birth/death gene turnover will likely be greatest for large gene families. Though EGC is expected to occur in such cases, disentangling EGC and gene turnover requires information about the age of individual members of a gene family, and polymorphism data to estimate the rate of EGC. Such data sets are difficult to obtain for large, repetitive gene clusters where individual copies cannot easily be distinguished [80,81].

### 4.2. Ectopic Gene Conversion from a Genome-Wide Perspective

It is currently unclear how prevalent EGC is at a genomic scale. To date, there have only been three genome-wide studies that directly addressed this question in *Drosophila*. These studies utilize different methodologies, yield different results, and emphasize EGC between gene families of different ages and degrees of sequence divergence. The perspectives of these studies seemingly reflect different evolutionary questions regarding the interplay between duplication and EGC. One perspective is geared towards understanding how EGC might govern the evolutionary fates of young duplicates. The other emphasizes broad patterns of EGC and is less concerned with the relative age or size of the gene families. Despite their differences, these studies utilize partially overlapping distributions of gene family ages, and it is within this region of overlap where some of the more puzzling differences emerge.

Analysis of the long-term effects of EGC was carried out by Hahn *et al.* [82] and Casola *et al.* [83], using genomic sequence from multiple *Drosophila* species ([84]; Hahn *et al.* analyzed gene families from 12 species' genomes; Casola *et al.* analyzed previously defined paralog pairs from 9 of 12 genomes). Hahn *et al.* applied maximum likelihood methods to infer rates of gene gain and loss along each branch of the species tree and then compared these results with those from a gene-tree/species-tree reconciliation analysis. If EGC has played a major role genome-wide, they expected that their reconciliation methods would infer multiple, parallel duplications across lineage. They estimated that approximately 17 genes were gained or lost every million years, with few signatures of EGC inferred by reconciliation analysis. The authors concluded that EGC leaves, at most, a minor genomic signature.

Casola *et al.* used GENCONV to estimate the proportion of genes in each species with evidence for EGC, and to assess whether different species exhibited different EGC rates. Relatively low estimates of EGC were inferred, with the proportion of converted paralog pairs ranging between 7.47% (in *D. melanogaster*) and 14.15% (in *D. grimshawi*). Peak conversion activity was observed for paralogs with silent divergence between d*S* = 0.1 and d*S* = 0.3. Phylogenetic reconciliation methods were also consistent with low amounts of EGC, with 1% to 3% of gene trees within the *D. melanogster* subgroup, and up to 15% of gene trees within deeper branches of the *Drosophila* tree, showing signs of EGC. The authors concluded that EGC was relevant for relatively young duplicates having silent divergence ranging between 0.1 and 0.3. Casola *et al.* also reapplied the likelihood-reconciliation methods used by Hahn *et al.* [84], and again found little support for EGC.

Osada and Innan [22] focused on the potential role of EGC during early duplicate evolution, and found widespread evidence for it. They restricted their analysis to duplication blocks within the sequenced genomes of *D. melanogaster*, *D. simulans*, *D. sechellia*, *D. yakuba*, and *D. erecta*, to identify duplication events immediately prior to or following divergence between *D. melanogaster*, *D. simulans*, and *D. sechellia*. The motivation of this approach was based on the expectation that EGC should be more active and more easily estimated in young gene families. EGC was estimated with reconciliation (tree-based) methods (carried out using entire duplicated regions and also using a sliding window analysis to test for variation across the sequence), and site-specific tests (see above). Of 28 post-speciation blocks available for the tree-based analysis, 24 provided evidence of EGC in the *D. melanogaster* lineage, the *D. simulans* lineage, or in both. The sliding window approach identified at least one signature of EGC in every block. Likewise, the site-based test identified a signal of EGC in 29/30 pre-speciation blocks.

Discordant estimates of EGC between these studies are likely to stem from multiple causes. In addition to their different criteria of duplicate selection, Casola *et al.* note that additional differences might result from the failure of Osada and Innan to account for parallel duplications between species (rapid birth-and-death rate), which potentially generate gene trees mimicking those predicted under a conversion model. They highlight estimates of high copy number variation (CNVs; [85]) as supporting this possibility. Additional methodological differences between the studies might also account for their results. A recent simulation-based study examined the performance of four commonly used methods for identifying EGC: reconciliation methods using paralog and otholog gene trees, sliding-window gene tree contrasts along duplicate sequences, GENCONV, and tests based on shared polymorphism [86]. The authors observed that the statistical power of reconciliation and shared polymorphism methods were positively correlated with the true rate of EGC, while the other two methods decreased in power with increased EGC rate. The detection range for GENCONV was limited to intermediate levels of divergence, likely leading to a net underestimate of EGC among Casola *et al.*' entire set of paralogs. The implementation of reconciliation methods also varied between studies, and while the performance of the approach taken by Osada and Innan was examined by Mansi and Innan [86], no comparison has been made between it and the particular approach of Casola *et al.*

Recent genome-wide estimates of CNV show that they are pervasive ([85,87,88]; in agreement with inferences based on comparative genomic studies: e.g., [89,90]). As Casola *et al.* note, these data suggest a high copy number mutation rate, which may drive a high rate of birth-and-death evolution. Because studies of concerted evolution deal primarily with intact full-length genes, the number of complete-gene CNVs is of particular interest. Emerson *et al.* ([85]; after correcting for false positive and false negative rates) discovered 73 polymorphic duplications and 10 deletions encompassing complete genes. While this represents a minority of their dataset, it can potentially contribute to an overestimate of the rate of EGC for some gene families. Arguing against this, the paralogous alignments in Osada and Innan exhibit degrees of nucleotide divergence (often in the flanking edges) that is too high to be consistent with segregating CNVs. In addition, only two of Osada and Innan's post-speciation duplicates were found to be CNVs in Emerson *et al.*' data set [22]. For pre-speciation CNVs, a birth-and-death interpretation would require parallel duplications between species that share the same (or very similar) breakpoints. Currently available CNV data in flies is insufficient to examine this possibility. However if parallel duplications have occurred between closely related species (evolutionarily young parallel duplicates), shared breakpoints should be detectable. To our knowledge, no such examples have been reported. Nevertheless, the amount of parallel duplications required to account for the disparate results of Osada and Innan [22] and Casola *et al.* [83] would likely have to be substantial.

## 5. The Genomic Context of Duplication and Ectopic Gene Conversion

A complete appreciation of EGC will require a deeper understanding of the genomic context in which it is most and least likely to occur, including simple factors such as DNA base composition (e.g., GC content) as well as complex factors, such as the three-dimensional conformation of chromosomes [91]. While there has been some effort to elucidate genomic features affecting EGC in *Drosophila*, there are currently *more* unresolved questions than answers.

The growing availability of genomic data has shed some light on features correlated with EGC. One tractable question is how the physical distance between duplicates correlates with EGC. Several studies indicate a negative correlation between physical distance and conversion between paralogs (data are based on case studies from *D. melanogaster* and more distantly related species: e.g., [75,78,92]). This pattern appears to hold for gene families dispersed across chromosomes, with paralog pairs on the same chromosome arm exhibiting stronger signals of conversion than pairs between chromosome arms [83]. This relationship between physical distance and EGC makes intuitive sense given the double-strand break model of gene conversion: following DSB in one duplicate copy, the initiation of a nonhomologous DNA repair pathway via the other paralog is more likely if the pair is in close proximity. Nevertheless, it is unclear whether the physical location in terms of a linear chromosomal map corresponds to actual “conversion proximity” in the context of a three-dimensional nucleus. Available analyses support the idea that chromosomal proximity facilitates EGC. However, this conclusion is tentative, given the typically conservative methods used to detect EGC (e.g., based on inter-paralog divergence data rather than more powerful polymorphism-based estimates) and the heterogeneous set of paralogs used in these studies (e.g., case studies and/or collections of duplicates of variable age).

Another question is whether EGC is biased. To date, there is no compelling evidence for this, yet the subject warrants future study. GC-biased conversion is often observed in cases of allelic (non-ectopic) conversion in mammals [93]. GC content in *Drosophila* duplicates is generally higher within conversion tracts relative to sequences that flank each tract, which suggests that the underlying conditions favoring conversion biases may commonly be present in paralogs [83]. Interestingly, when converted paralogs were compared with noncoverted paralogs belonging to the same family, GC content was not higher within converted relative to uncoverted regions. These patterns suggest that nucleotide composition might promote EGC rather than biasing the direction of conversion toward GC nucleotides [83].

## 6. Interaction between Selection, Ectopic Gene Conversion and Evolutionary Divergence between Paralogs

The interaction between EGC and natural selection is central to interpretations of concerted evolution patterns, as well as inferences about the rate of ongoing EGC. The likelihood of conversion between non-allelic sequences is, in part, a function of their degree of sequence identity. For gene family members evolving under strong purifying selection, relatively high sequence identity is expected in the absence of EGC. EGC will further reduce divergence between paralogs by homogenizing (putatively) neutrally evolving synonymous sites, introns and intergenic DNA, and by promoting parallel adaptation in functionally relevant sites (e.g., nonsynonymous or regulatory DNA). For example, the interaction between EGC and natural selection may prevent the accumulation of deleterious mutations [81,94–96], or facilitate the spread of new beneficial alleles among gene family members [97].

While EGC can promote adaptation among functionally redundant genes, it may also constrain adaptive differentiation between paralogs—a process that might impact the evolution of new gene functions [17,98–100]. The homogenizing effect of EGC is expected to limit opportunities for “neo-functionalization”—the evolution of novel functions among young duplicates [18,23,101–103].

Some evidence from *Drosophila* supports both reinforcing and antagonistic interactions between selection and EGC. Positive selection between paralogs has been observed in several general contexts [76,104,105]. Some instances of positive selection also apply to gene families that are simultaneously experiencing EGC [22,75,79]. These latter studies suggest that EGC and selection occasionally come into conflict with one another. For example, if EGC and selection occurred simultaneously, the observed molecular signatures of adaptation indicate that selection was strong enough to counteract the process of homogenization. On the other hand, evidence that EGC overwhelms disruptive selection is unlikely to be detected on a case-by-case basis, because paralog homogeneity will be consistent with both strong EGC relative to selection, or with a lack of disruptive selection.

Conflict between EGC and disruptive selection might potentially be examined by comparing patterns of divergence between paralog pairs experiencing markedly different rates of EGC. For example, if paralogs on different chromosomes experience reduced EGC compared to closely linked paralogs (as appears likely in *Drosophila:* e.g., [78,83,92,105]; but see [79]), one might systematically test for signatures of positive selection between duplicates within *versus* between chromosomes. Thornton & Long [104] compared inter- and intra-chromosomal paralog divergence throughout the *D. melanogaster* genome, and found a pattern of increased divergence (Ka/Ks) when both duplicates resided on the X chromosome, but otherwise no consistent effect of intra- *vs.* inter-chromosome paralog orientation. To the extent that amino acid divergence has been driven by positive selection, the pattern does not indicate any constraint imposed by EGC.

There are two important caveats associated with such contrasts between linked and unlinked duplicates. First, any constraint imposed by EGC is expected to primarily occur during the early evolutionary divergence of paralogs, yet most duplicate genes are relatively ancient. Even if EGC provided constraint during the early evolution of duplicates, its signature will often be erased by sequence divergence subsequent to the cessation of EGC. Furthermore, retention of ancient duplicate genes might imply that they have evolved an important biological function, for which they are maintained. Ancient duplicates may therefore be enriched for genes that have “overcome” the effect of EGC to evolve non-redundant functions (e.g., neo- or sub-functionalization); these ancient duplication events may represent a filtered (and therefore biased) set of duplicates. Osada and Innan [22] have emphasized the utility of using young gene duplicates to study the relative roles of EGC and selection during the early, “fate-determining” stage of paralog evolution. In their dataset of young duplication events, they observed widespread signatures of EGC, including an apparent case of adaptive paralog differentiation in the face of concerted evolution. Analysis of young duplications represents a powerful means to address the potential conflict between selection and EGC. Deep resequencing efforts that are currently underway in *D. melanogaster* should enhance statistical power to identify and estimate the rate of EGC, particularly in small gene families or other low-copy repeat sequences. Polymorphism data will also permit discrimination between evolutionary models of genetic drift and positive selection (this latter goal may require an extension of MK-based statistical tests of positive selection, which currently apply to independently evolving orthologs [106,107], rather than gene families undergoing some degree of EGC).

The second caveat concerns the potential relationship between selection and degree of dispersion between duplicates. While a negative relationship between EGC rate and distance is expected (see above), it is also possible that disruptive selection between duplicates might also covary with distance. If, for example, unlinked paralogs are exposed to different local chromatin states, or are influenced by distinct local promoter sequences, then the opportunity for disruptive selection might increase with greater dispersion between paralogs. Creative statistical and bioinformatic approaches will be required to control for possible spurious correlations between distance and adaptive differentiation.

## 7. Temporal Dynamics of Duplication and Paralog Divergence

EGC can influence both the temporal patterns of duplicate gene evolution, and interpretations of these patterns within the context of evolutionary theory. The inference of selection and genetic drift from empirical properties of duplicate genes (*i.e.*, their ages and the distribution of inter-paralog divergence) will critically depend on whether or not EGC occurs between paralogs, as well as the long-term covariance between selection, EGC, and paralog divergence. Our understanding of the actual dynamics of EGC can have a major impact on our interpretation of: (1) the rates of duplicate birth and death, and the age distribution of duplicate genes and (2) the temporal patterns of selection during the course of duplicate evolution.

The inferred rate of gene duplication is sensitive to assumptions about the degree of evolutionary independence between paralogs. Without EGC, neutral sequence divergence between duplications (e.g., the number of synonymous site differences, or d*S*, between them) should be clock-like, and proportional to the relative age of the duplication event. EGC will downwardly bias the distribution of d*S*, and lead to an overestimation of the duplicate “birth” and “death” rates (high rates of origin and loss will also skew the age distribution toward young duplicates). However, since birth-and-death and EGC rates are largely unknown, the distribution of d*S* is insufficient for inferring the evolutionary rate and maintenance of gene duplicates. Exploiting genome sequence data from closely related species can circumvent this methodological limitation. Osada and Innan's [22] identification of 31 young duplications since the *D. melanogaster/D. simulans* last common ancestor (about 2.3 million years ago) suggests a duplication rate of approximately 10^−9^, per gene, per year, which is approximately ten-fold lower than earlier estimates based entirely on d*S*. Given the relatively short time interval separating these species (and small number of duplication events), this estimate may differ from the true duplication rate in Drosophila, yet it should characterize the rate to an order of magnitude.

Another common observation is a negative relationship between the ratio of nonsynonymous to synonymous divergence between paralogs (*i.e.*, d*N*/d*S*) and the silent substitution rate (d*S;* [108]). d*N*/d*S* is often used as a metric of evolutionary constraint, with values close to zero associated with strong purifying selection at nonsynonymous sites, and larger values associated with some combination of neutral and adaptive divergence. Assuming there is no conversion between young paralogs, the negative relationship between d*N*/d*S* and d*S* suggests that evolutionary constraint is (on average) stronger for ancient relative to young duplicates (with data based on multiple taxa, including *Drosophila*; [108,109]): young duplicates either experience relaxed purifying selection or enhanced opportunities for adaptive differentiation. This relationship can be exacerbated when “young” duplicates (those with high sequence identity) experience higher rates of EGC than ancient duplicates. EGC is generally expected to reduce d*S* between paralogs, and will similarly reduce d*N* if nonsynonymous substitutions are also evolving neutrally. If nonsynonymous substitutions are being driven by differential positive selection between paralogs, then EGC is expected to more strongly depress d*S* relative to d*N* (and upwardly bias d*N*/d*S*), and the correlation between d*N*/d*S* and d*S* may become more strongly negative.

## 8. Conclusions

EGC can profoundly influence the evolutionary fates of young duplicates, as well as the patterns of concerted evolution within gene families of varying size and age. In *Drosophila*, evidence for EGC is particularly strong in small gene families (e.g., of size two) with high sequence identity between paralogs (on a case-by-case basis, this might be due to strong evolutionary conservation of duplicates, or to their recent origin). For larger gene families, and/or ancient paralog pairs, evidence for EGC is weaker, and is often difficult to distinguish from birth-and-death models.

Methodological limitations preclude a precise estimate for the rate of EGC, and are expected to cause a statistical bias towards type II error (by failing to detect EGC, even though it is occurring). The growing feasibility of collecting and analyzing whole-genome polymorphism datasets (already underway within Drosophila; [110]) will soon help to remedy this issue. Polymorphism-based methods greatly increase the power to detect EGC, and polymorphism-oriented statistical methods have already been developed for estimating rates of EGC.

The confluence of three sources of data—improved EGC estimates, rapidly accumulating CNV data that can be used to infer mutational processes for duplicates, and multispecies phylogenies (e.g., the 12 *Drosophila* genomes and beyond) for calculating the ages of gene family members—should soon favor an increasingly sophisticated analysis and interpretation of the evolutionary consequences of EGC, including its interaction with mutation, selection, and linkage.
